# Small Molecule Inhibitors in Adult High-Grade Glioma: From the Past to the Future

**DOI:** 10.3389/fonc.2022.911876

**Published:** 2022-06-17

**Authors:** Wenda Huang, Zhaonian Hao, Feng Mao, Dongsheng Guo

**Affiliations:** ^1^Department of Neurosurgery, Tongji Hospital, Tongji Medical College, Huazhong University of Science and Technology, Wuhan, China; ^2^Department of Neurosurgery, Beijing TianTan Hospital, Capital Medical University, Beijing, China

**Keywords:** glioblastoma, small molecule inhibitor, molecular mechanism, TKI—tyrosine kinase inhibitor, clinical trial

## Abstract

Glioblastoma is the most common primary malignant tumor in the brain and has a dismal prognosis despite patients accepting standard therapies. Alternation of genes and deregulation of proteins, such as receptor tyrosine kinase, PI3K/Akt, PKC, Ras/Raf/MEK, histone deacetylases, poly (ADP-ribose) polymerase (PARP), CDK4/6, branched-chain amino acid transaminase 1 (BCAT1), and Isocitrate dehydrogenase (IDH), play pivotal roles in the pathogenesis and progression of glioma. Simultaneously, the abnormalities change the cellular biological behavior and microenvironment of tumor cells. The differences between tumor cells and normal tissue become the vulnerability of tumor, which can be taken advantage of using targeted therapies. Small molecule inhibitors, as an important part of modern treatment for cancers, have shown significant efficacy in hematologic cancers and some solid tumors. To date, in glioblastoma, there have been more than 200 clinical trials completed or ongoing in which trial designers used small molecules as monotherapy or combination regimens to correct the abnormalities. In this review, we summarize the dysfunctional molecular mechanisms and highlight the outcomes of relevant clinical trials associated with small-molecule targeted therapies. Based on the outcomes, the main findings were that small-molecule inhibitors did not bring more benefit to newly diagnosed glioblastoma, but the clinical studies involving progressive glioblastoma usually claimed “noninferiority” compared with historical results. However, as to the clinical inferiority trial, similar dosing regimens should be avoided in future clinical trials.

## Introduction

Glioblastoma (GBM) is the most common primary malignant brain tumor. Based on the data from 2011 to 2015 in the United States, the average annual age-adjusted incidence of GBM is 3.21 per 100,000 population, with an overall prevalence of 9.23 per 100,000 population (1). Maximal safe surgical resection followed by radiotherapy with concomitant and adjuvant temozolomide has gradually become the standard regimen since 2005 (2). However, after standard therapy, almost every patient recurs within 6 months, and there is no effective therapy for recurrent GBM (3, 4). Although plenty of effort has been made toward studying the mechanisms of pathogenesis and progression of glioma, there was no significant change in treatment regimen and survival for GBM patients. In the past 20 years, targeted cancer therapies were promising methods, and simultaneously, various targeted drugs gradually entered clinical trials and then were approved by the FDA for cancer treatment.

**Figure 1:**
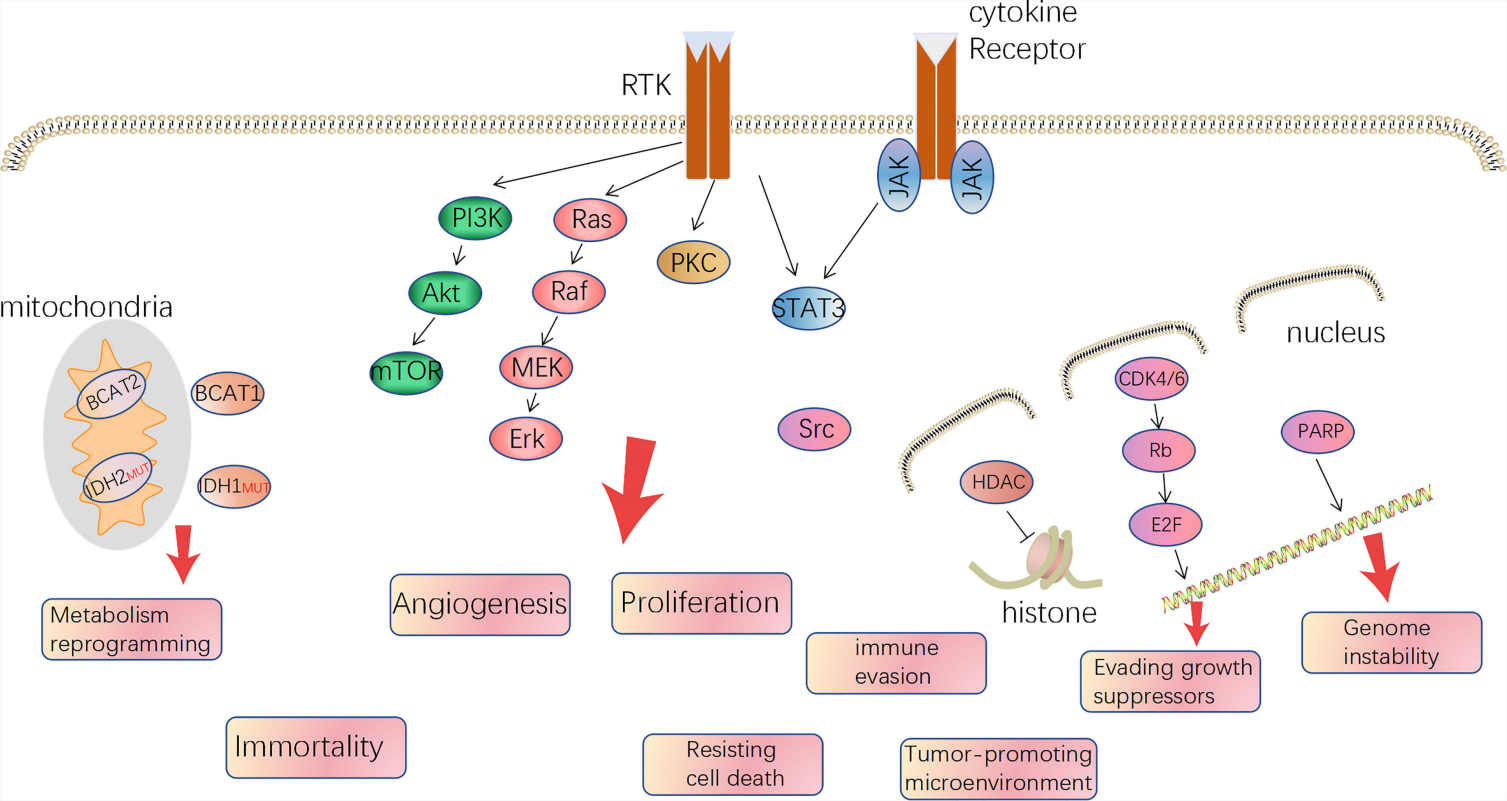
The major druggable targets in glioblastoma and corresponded hallmarks of tumor.

Gliomas were deemed to derive from neural stem cells and, based on different classification criteria, can be generally divided into circumscribed gliomas and diffuse gliomas, or into low-grade (WHO I, II) and high-grade (WHO III, IV), or adult-type and pediatric-type (5–7). Futhermore, adult high-grade diffuse glioma affects the most people and is also the hardest to cure, predominantly including GBM (WHO IV) and anaplastic glioma (AG, WHO III, including AOD: anaplastic oligodendroglioma and AA: anaplastic astrocytoma) and accounts for approximately 80%. In 2008, Parsons et al. computed by the TCGA database and proposed three core alterations of signaling pathways in GBM: P53, retinoblastoma pathway (pRB), and receptor tyrosine kinases (RTKs) (8). Besides, mutation of phosphatase and tensin homolog deleted on chromosome ten (PTEN), neurofibromatosis (NF1), isocitrate dehydrogenase (IDH), B-Raf Proto-Oncogene (BRAF), and chromosome 1p19q co-deleted are common in glioma, even being used for molecular classification and prognosis prediction. In 2016, it was the first time the WHO Classification of CNS Tumors used molecule profiles to define the sub-types of gliomas. It is an important way to translate the abundant knowledge of the moleculer mechanisms to clinical applications (9). But regardless of IDH mutation, 1p/19q co-deletion, and H3 K27 mutation, the therapy regimen is similar and the classification method plays a limited role in guiding treatment. On the other hand, all the typical hallmarks of cancer can be detected in GBM, especially angiogenesis, tumor-promoting immune microenvironment, and reprogramming metabolism, which is gradually getting attention (10). The relative mechanisms of the hallmarks become therapeutic targets (**Figure 1**). Over the last two decades, since the specimen which was capable of generating tumor cells containing multiple lineage markers was reported, the concept of glioma stem cells (GSCs) has been more and more accepted by scientists (11, 12). GSCs are ascribed to a population accounting for heterogeneity in glioma mass and therapeutic resistance through self-renewal and differentiation to variant subpopulations. The elucidation of features of GSCs may be a key step in developing next-generation therapies (13). However, GSCs were not a bulk of homogeneous cells but rather a mosaic of discrete populations with distinct features (14). The complexity of GBM requires us to more deeply understand the mechanisms, in particular, the genetic and phenotypic characteristics of subclones distributed in the mass of tumor (15, 16).

**Figure 2:**
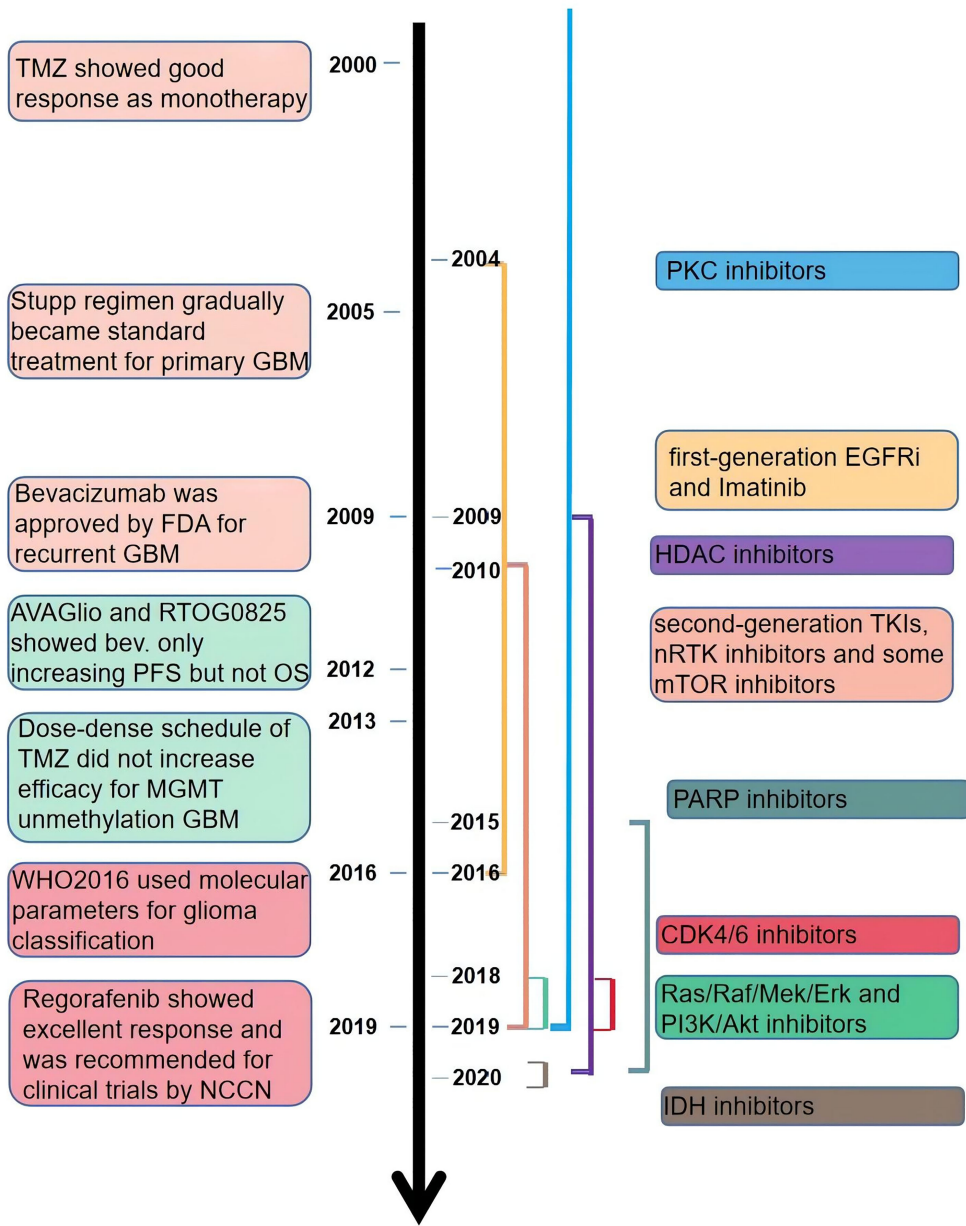
The time line of publicly published clinical trials of the small-molecule inhibitors.

NCCN guidelines on central nervous system cancers indicate that the preferable treatment option for patients with recurrent GBM is enrollment in clinical trials due to the dismal outcomes of other therapies, including carmustine/lomustine, TMZ, radiotherapy, and bevacizumab (Bev.). There are more than 1,500 clinical trials associated with glioma completed or ongoing, among which contain hundreds of targeted therapies (https://clinicaltrials.gov/, **Figure 2**). Targeted therapies predominantly include two methods: small molecule and monoclonal antibodies. Simultaneously, various gene therapies such as microRNA, lncRNA, and exosome are a hotspot of research. Compared with monoclonal antibodies, small-molecule inhibitors possess several exclusive characteristics: reduced financial burden, taken orally, and more extensive druggable targets, including intracellular proteins (17). According to the targets of the small molecule inhibitors, we divided them into three main categories: tyrosine-kinase inhibitor (TKI), non-receptor tyrosine kinase (nRTK), intracellular signal transduction pathway inhibitor, and cellular biological process inhibitor. TKIs are still the most studied small-molecule inhibitors. But the outcomes of TKIs for the treatment of GBM and AG are often disappointing. An important reason for this is the complex network of intracellular signaling pathways leading to drug resistance. Besides, interference with biological processes and impact on the hallmarks of tumor growth can inhibit the growth of tumors through different mechanisms. In this review, we revisited the outcomes of recent clinical trials of adult high-grade glioma (mainly including GBM and AG) published in PubMed and the functional mechanisms of small-molecule inhibitors (**Additional File 1**). We hope to provide advice on the combination of drugs in future clinical trials by understanding the characteristics of different drugs.

## RTK

**Figure 3:**
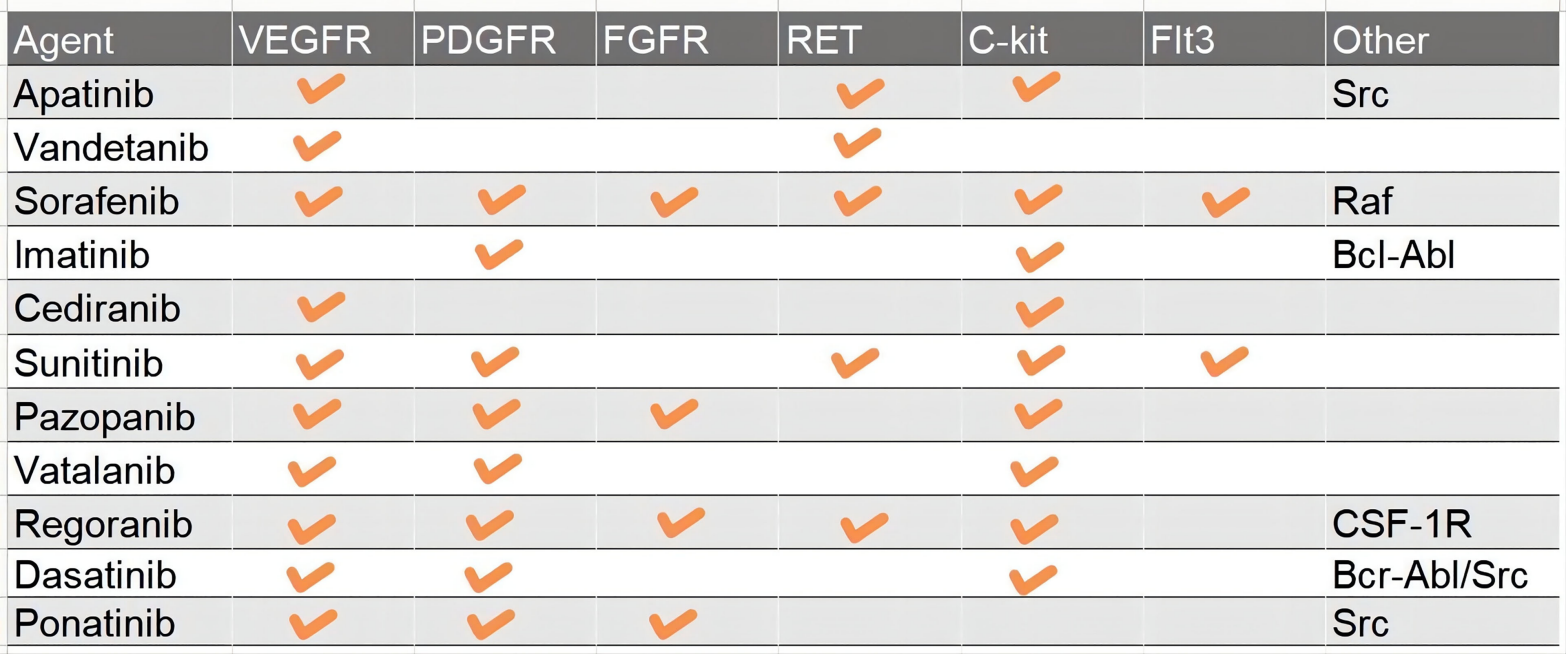
**A** table which summary the major targets of Pan-TKIs.

Receptor tyrosine kinases (RTKs) and the downstream signal transduction are the most characterized networks associated with glioma pathogenesis and progression. To date, scientists have found more than 60 RTKs such as EGFR, VEGFR, MET, PDGFR, and FGFR (18, 19). Mutations in RTKs are also the most frequent alterations in GBM, namely, copy number variation, structure variation, nucleotide variation, and over-activation of the autocrine growth factor/receptor loop (18, 20). And then RTKs cause deregulation of several important pathways, namely, Ras/MAPK, PI3K/Akt/PTEN, PLC gamma/PKC, and Jak/STAT3 (21, 22). For instance, more than 50% of GBM harbor EGFR amplification and 25% cause EGFR mutation (23). EGFRvIII, a common mutant of EGFR which is continually activated even without ligand binding to the receptor, occurs after the pathogenesis of GBM to change the pattern of tumor growth and increase the intra-tumoral heterogeneity and resistance to targeted therapies (23). PDGFR, VEGFR, and FGFR are the most important RTKs associated with tumor angiogenesis (24). c-Met, also known as mesenchymal–epithelial transition factor, plays an important role in migration, therapy resistance, and vasculogenesis. In addition, Ret, c-Kit (CD177), and Flt3 (CD135) are also the targets of TKIs. The dynamic change of variant RTKs over time and space in tumor entities accounts for the complexity of GBM and therapy resistance after recurrence (3). RTKs also regulate the expression of GSC relevant transcription factors such as OLIG2, SOX2, and ZEB, which are also GSC marker molecules (25, 26). According to the chronological order, TKIs include three categories, and up to now, the most studied TKIs in glioma are predominantly first and second generation. The first-generation EGFR inhibitors, also known as type I, including erlotinib and gefitinib, can reversely bind to the ATP-binding site of EGFR to inhibit the activity of the receptor (27, 28). The second-generation inhibitors, such as afatinib, neratinib, vandetanib, and dacomitinib, irreversibly inhibit EGFR. Besides EGFR, the second-generation inhibitors can often inhibit multiple other RTKs, such as Her2, PDGFR, MET, and VEGFR (29–32). The third-generation inhibitor, Osimertinib, is used to overcome the resistance due to the T790M mutation, which is usually acquired after treatment with first-generation EGFR inhibitors. But researchers found that the acquired exon 20 C797S mutation would lead to resistance to Osimertinib within 10 months (33). So, the next-generation inhibitor, such as EAI045, is proposed to solve the problem of T790M mutation and C797S mutation inducing resistance (33). sorafenib, anlotinib, cabozantinib, cediranib, imatinib, lenvatinib, sunitinib, pazopanib, and motesanib enter clinical trials in glioma after approval by the FDA for renal cell carcinoma, lung cancer, thyroid cancer, and other solid tumors. They can target PDGFR, FGFR, C-kit, MET, and some nRTKs, which play great roles in the growth of GBM (**Figure 3**).

Until June 2021, there have been approximately 85 clinical trials of diverse TKIs to treat GBM and AG published in PubMed, including 17 studies for newly diagnosed GBM and 68 studies for progressive GBM. When comparing the results extracted from the publication with the baseline (historic results of 9 important clinical trials), there are 27 of 40 studies that show improvement in radiological response rates (ORs). These studies mainly involved patients with recurrent or progressive GBM. PFS was evaluated as mPFS in 71 studies and as PFS6 in 61 studies. There were 6 of 17 studies involving newly diagnosed GBM and 13 of 63 studies involving recurrent GBM that had an improvement in mPFS compared with baseline. OS was evaluated as mOS in 72 studies and as OS12 in 22 studies. But the most urgent problem is the only improvement of PFS while infrequently increasing the OS (**Additional File 1**).

Erlotinib is the most studied TKI in glioma. A phase II trial in 2014 that added Bev. and erlotinib to TMZ after completion of radiation involving 59 patients with newly diagnosed GBM showed an amazing result (mPFS: 13.5 m; mOS: 19.8 m), although the study did not reach the primary endpoint of improved OS (34). A study in 2010 using erlotinib as monotherapy for recurrent GBM had 40% PFS6 and 53% OS12 with 14 months of mOS (35). In summary, regardless of monotherapy or combined regimen, the result did not reach the expected outcome. And the improvement of ORs was partly deemed to be unauthentic because of improper imaging criteria (McDonald criteria). Combining erlotinib with other small-molecule inhibitors, such as sorafenib and sirolimus, did not play a greater role. Maybe due to the toxicity of sirolimus and its derivate temsirolimus, erlotinib combined with sirolimus decreased the PFS and OS in a phase II trial for recurrent malignant glioma (36, 37). Sorafinib is a pan-TKI, and it can also effectively inhibit the Ras pathway. Based on hypothesizing that inhibition of both EGFR and Ras would improve the survival time of patients with recurrent GBM, a phase II trial in 2013 was launched but did not reach the effective hypothesis H0 (30% increase in overall survival time compared with historical controls) (38). More clinical trial results will be helpful to clarify the efficacy of the combination of sorafenib and erlotinib. To date, the studies of erlotinib in glioma have stopped in phase II trials, and there is not enough evidence to show the potential efficacy of erlotinib.

**Figure 4:**
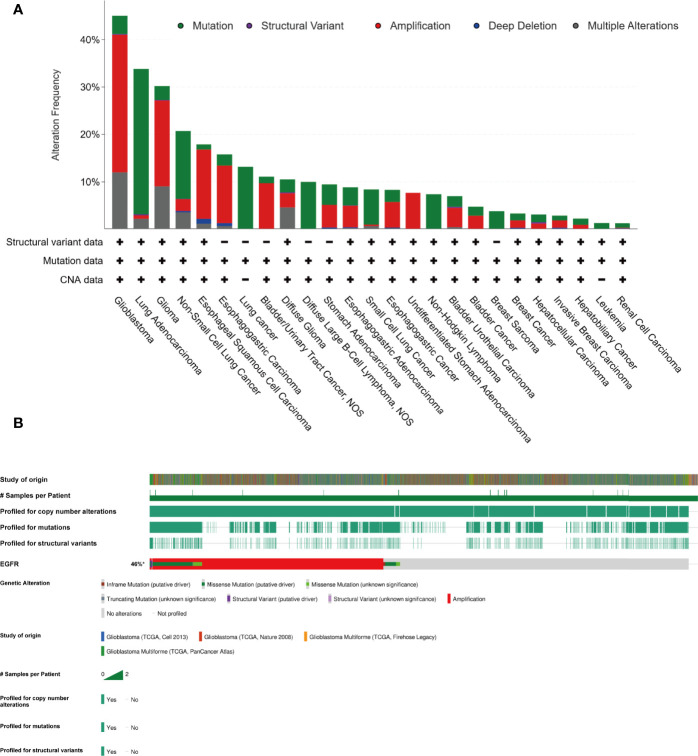
In contrast to lung cancer, glioma majorly harboring amplification of EGFR, instead of nucleotide mutation (cBioPortal). **(A)** The EGFR alteration frequency in different tumor types based on the PanCancer study “Pan-cancer analysis of whole genomes (ICGC/TCGA, Nature 2020)” in cBioportal database. **(B)** The collection of genetic alteration of EGFR based on four different studies in cBioportal database.

Gefitinib is another well-characterized first-generation EGFR inhibitor. Just like erlotinib, it is more effective in cancers with mutated and overactive EGFR. Gefitinib did not show the potential to increase the OS of glioma. In 2012, a phase I/II trial involving 178 newly diagnosed GBM combining gefitinib with radiotherapy showed a disappointing result (mPFS: 4.9 m, mOS: 11.5 m) compared to baseline (38). Two important trials using gefitinib monotherapy for recurrent CNS tumor (NCT00025675, 2003) or combination with radiation for newly diagnosed GBM (NCT00052208, 2003) involving 105 and 158 patients respectively did thus far not show their results. There are no more results to evaluate the efficacy of gefitinib. As such, the outcomes of two classic first-generation EGFR inhibitors are a little disappointing. The insensitivity of GBM to erlotinib and gefitinib is partly due to the different models of EGFR overactivity in GBM cells. The two first-generation TKIs have a higher inhibiting ability of mutant EGFR such as L858R and exon 19 deletion type EGFR, which increases the kinase activity. In contrast to non-small cell lung cancer and adenocarcinoma harboring activating nucleotide mutations within the EGFR kinase domain, GBM overly activates EGFR by the increasing copy number and the substituted mutations occur in the extracellular domain (39) (**Figure 4**).

Lapatinib can selectively inhibit EGFR and HER2. Lapatinib combined with TMZ is a potential salvage option for recurrent ependymoma due to a phase II study (NCT00826241) demonstrating clinical activity with objective responses and prolonged disease control associated with disease-related symptom improvements (40). To date, three clinical trials including 74 patients did not present good results (41–43).

Afatinib is a second-generation irreversible ErbB family inhibitor, mainly used to treat cases of NSCLC that harbor mutations in the EGFR gene. Simultaneously, afatinib can effectively block EGFRvIII, a common mutant occurring in glioma (23, 44). A phase I study (NCT00977431) of afatinib combined with chemoradiotherapy demonstrated a favorable objective rate but simultaneously resulted in a high rate of serious adverse events. In 2015, a parallel-arm phase II trial (NCT00727506) showed that the addition of afatinib significantly decreased the PFS6 compared with TMZ alone (44). In the future, a pulsatile-increased dosing schedule of afatinib will be tested in glioma. It may be feasible to make a difference and reduce the adverse effects (44).

Vandetanib acts as a kinase inhibitor of several receptors, including VEGFR, EGFR, and the RET-tyrosine kinase. Because of the dual inhibition of EGFR and VEGFR2, vandetanib was supposed to play a greater role than combining erlotinib and Bev. in inhibiting angiogenesis. The prevailing rates and PFS usually showed favorable increases. A phase I/II study (NCT00441142) of parallel arm design demonstrated that adding vandetanib to TMZ and radiation can modestly increase the OS (45). These results give researchers the faith to continue their clinical studies. There are more than 7 phase I studies ongoing.

Sorafenib is a classical pan-TKI, targeting VEGFR, PDGFR, FGFR, c-Kit, Flt3, and RAF kinases, which are important in the angiogenesis of solid tumors. Pan-TKI is a trend of development of small-molecule inhibitors because of the complex crosstalk between various growth factors and compensatory mechanisms of RTKs. GBM cells can rapidly switch their signaling addiction from reliance on a single RTK to multiple RTK pathways. This may be driven by glioma stem cells (46). In general, sorafenib did not demonstrate sufficient efficacies to improve the outcome of patients with glioma. Many phase II trials (NCT00597493, NCT00544817, and NCT00621686) that have added sorafenib into the Stupp protocol or Bev. showed disappointing outcomes, even those inferior to Bev. monotherapy (47–49). In addition, sorafenib combined with erlotinib can play a synergic role by inhibiting EGFR and other RTKs in theory. But a phase II study (NCT00445588, 2013) using the combination regimen only showed an OS of 5.7 months and a PFS6 rate of 14% (38). Sorafenib combined with mTOR inhibitors such as temsirolimus did not improve the outcome in two phase I/II studies (NCT00335764, NCT00329719). Many clinical trials evaluating targeted combinatorial blockade frequently have disappointing outcomes due to overlapping toxic events, including serious diarrhea, seizure, and rash. Alternative administration schedules will probably reduce toxicity (17).

Imatinib is a specific inhibitor of several tyrosine kinase enzymes, including ABL, c-Kit, and PDGFR by occupying the tyrosine kinase active site to decrease the activity of kinases. PDGFR is deemed as a driven gene in low-grade glioma and is overexpressed in GBM. Imatinib has limited efficacy as monotherapy in GBM, and the chemotherapeutic agent hydroxyurea (HU) was found to play a synergic role when combined with imatinib due to imatinib sensitizing cancer to chemoradiotherapy and the penetrability of the blood–brain barrier (BBB) of HU (50). A multi-center phase II study (NCT00290771) involving 231 patients with progressive GBM showed a good safety profile among patients, which receiving up to 1,000 mg of imatinib and 1,000 mg of hydroxyurea. But the efficacy is not enough to induce anti-tumor (51). A phase III study (NCT00154375) involving 240 patients with TMZ-resistant GBM presented similar results of not meeting the primary study end point despite a good safety profile (52). Thus far, imatinib has not brought good news for glioma patients, just like in CML.

Cediranib is mainly a VEGFR inhibitor, and later the inhibiting c-Kit was found. Cediranib had a narrower inhibiting spectrum of RTKs than other pan-TKIs. But just because of this, cediranib has the chance to function as anti-VEGF therapy in combination with chemoradiotherapy for newly diagnosed GBM (NCT00662506 in 2008; NCT01062425 in 2010). The adverse effects of multiple TKIs are predominantly driven by the inhibition of VEGFR (17). The anti-angiogenesis and normalization of micro-vessels driven by anti-VEGF such as Bev. are deemed to sensitize patients to radiotherapy by increasing the supply of oxygen (53). The impact of angiogenesis inhibition on tumor distribution of TMZ is also a key factor considering the combination of standard therapy and anti-angiogenesis drugs (54).

Sunitinib inhibits all subtypes of PDGF-Rs and VEGFRs and c-Kit, RET, so that the safety is disquieting (55). Recently, sunitinib has mainly functioned as a monotherapy in clinical trials of CNS tumors, but the outcomes of patients were dismal. A phase II study (NCT00923117) of sunitinib to treat recurrent brain cancer has been terminated because of an unexpected result. The OS of Bev. resistant patients is 0.92 months and that of Bev. naive patients is 1.08 months. The regimen of sunitinib monotherapy caused high adverse event rates of 100% with more than 30% serious adverse events, such as skin, mucosal, and gastrointestinal adverse events (56). Bev. is a unique targeted therapy drug recently approved by the FDA for recurrent GBM. The various TKIs are considered in combination with Bev. or to be used in Bev.-resistant patients, even the VEGFR inhibitors. Continuous schedules enhance the efficacy of sunitinib. How to balance the dose plan and safety is the key factor to acquiring a satisfactory outcome.

Pazopanib limits tumor growth by targeting angiogenesis *via* the inhibition of VEGFR, PDGFR, c-KIT, and FGFR (57, 58). A phase II study (NCT00459381) involving 35 patients with recurrent GBM was disappointing (59). Adding chemotherapeutic drug topotecan with pazopanib did not significantly increase the OS of patients with recurrent GBM (NCT01931098) regardless of prior Bev. exposure or not. Simultaneously inhibition of EGFR and other RTKs increased the efficacy and theoretically led to a series of phase I trials to evaluate the safety and maximum toxic dose of the combination of EGFR inhibitors and pazopanib (60–62). But the accurate efficacy of GBM warrants more studies.

Vatalanib was designed for advanced cancers, especially those that have not responded to chemotherapy. It inhibits all known VEGF receptors, as well as PDGFR-beta and c-kit, but is most selective for VEGFR-2 (63). The outcomes of two phase I trials for newly diagnosed GBM were not poor compared with baseline, but the efficacy of vatalanib for treating GBM needs to be tested in more clinical trials.

Regorafenib, as a sorafenib derivative and pan-TKI of several kinases involved in tumor angiogenesis (VEGFR1–3 and TIE2), oncogenesis (KIT, RET, RAF1, and BRAF genes), the tumor microenvironment (PDGFR and FGFR), and tumor immunity (colony-stimulating factor 1 receptor), is recommended in the newest NCCN guideline for testing in future clinical trials due to a phase II parallel trial harboring a notable outcome (64, 64). In addition, regorafenib can induce autophagy arrest by the PSAT1/PRKAA autophagy-initiating pathway (65). By 2015, it had two US approvals for advanced cancers. However, the conclusion from the phase II study was defective. The outcome of regorafenib was just better than that of the control arm in the trial instead of baseline (66).

Many other TKIs are being tested in clinical trials, namely, anlotinib, cabozantinib, dovitinib, vatalanib, tandutinib, lenvatinib, and nintedanib, but so far, there are few trials with outcomes of these small-molecule inhibitors. Some TKIs have been approved by the FDA for other types of cancer and have not been widely tested in glioma. For instance, third-generation osimertinib with improved CNS penetration has broader coverage for mutated versions of EGFR.

In summary, (i) first EGFRi such as gefitinib and erlotinib, and PDGFRi imatinib may play great roles in specific patients with over-acting EGFR (especially EGFR with kinase domain mutation) and PDGFR. However, Pan-TKIs, such as sorafenib, sunitinib, and regorafenib did not show enough efficacy thus far (**Table 1**). (ii) Afatinib, which block EGFRvIII, a common structure mutation occurring after over-activating wild-type EGFR. The judgment of the time window of afatinib is a challenge of deeply understanding of heterogeneity in GBM temporally and regionally. (iii) VEGFR is a more important target than EGFR for GBM therapy. Anti-angiogenesis is a promising method including vandetanib, cediranib, vatalanib, and cabozantinib, just like Bev. Normalizing the immature vessels in the mass of glioma may play a supporting role in other therapies. (iv) Modest efficacy of inhibiting wild-type EGFR, effectively suppressing VEGFR, FGFR to anti-angiogenesis, along with inhibiting EGFRvIII may play an unexpected role. (v) Blindly using Pan-TKIs such as sorafenib, sunitinib, and regorafenib always did not show better efficacy and rather brought the risk of off-target effects. (vi) The side effects of TMZ and radiation are brain necrosis and myelosuppression, respectively. The side effects of TKIs are diarrhea and rash. Using TKIs and decreasing the dose of chemoradiotherapy may reduce the side effects, especially for patients over the age of 70 or patients with poor performance status to improve the life quality. (vii) The methylation status of the MGMT promoter is an important prognosis factor for TMZ therapy (Stupp regimen, MGMT methylated mOS 23.2 m, mPFS 10.5 m; MGMT unmethylated mOS 16.0 m, mPFS 7.8 m). As for MGMT unmethylated patients, TKIs may play an effective role. In the future, RTKs will still be the prime target for treatment of glioma because of their irreplaceable functions. To seriously appreciate the efficacy of various TKIs, we need more idealized randomized controlled trials just like AVAglio and RTOG 0825 for Bev.

**Table 1:** Part of clinical studies which claimed “non-inferiority” for historical results.

Small molecule	First author	Phase	Year	Patient number	Therapy	CR	PR	OR	SD	mPFS	PFS6	mOS	OS12
gefitinib	Amanda L. Schwer (67)	I	2008	15	gefitinib 250 mg pod + radiotherapy		13.3%	13.3%	13.3%	7 m	63.0%	10 m	40.0%
erlotinib	Jeffrey J. Raizer (35)	I	2010	32	erlotinib 150–775 mg pod	3.3%		3.3%	6.7%		~40%^1^	14 m	53.0%
	Jennifer L. Clarke (34)	II	2014	59	ertlotinib + TMZ + RT + bev.					13.5 m		19.8 m	
afatinib	David A. Reardon (44)	II(pilot)	2015	119	afatinib 40 mg pod	0.0%	2.4%	2.4%	34.1%		3.0%	9.8 m	
					afatinib 40 mg + TMZ	2.6%	5.1%	7.7%	35.9%		10.0%	8.0 m	
Vandetanib	Jan Drappatz (54)	I	2010	13	Vandetanib + TMZ+ RT				90.0%	8 m	~75%	11 m	~80%
Cabozantinib	Patrick Y. Wen (68)	II	2018	222	cabozantinib at up to 140 mg/day		17.6%	17.6%		3.7 m	22.3%	7.7 m	~35%
					cabozantinib at up to 100 mg/day		14.4%	14.4%		3.7 m	27.8%	10.4 m	~40%
Imatinib	David A. Reardon (50)	II	2005	33	imatinib mesylate 400–500 mg + hydroxyurea (500 mg twice a day)					3.4 m	27.0%	11.4 m	
	David A. Reardon (69)	I	2008	65	imatinib + TMZ					6.2 m	52.3%	11.1 m	
	David A. Reardon (70)	I	2009	37	vatalinib + imatinib + hydroxyurea		24.3%	24.3%	48.6%	3 m	25.0%	12 m	
sorafenib	David A. Reardon (47)	II	2011	32	sorafenib (400 mg twice daily) + temozolomide	3.1%		3.1%	46.9%	1.5 m	9.4%	14.3 m	34.4%
vorinostat	Katherine B. Peters (71)	I/II	2018	39	vorinostat 200–400 mg/d +TMZ + bev.	5.10%	38.50%	43.60%		6.7 m	53.80%	12.5 m	51.30%
veliparib	H. Ian Robins (72)	I/II	2016	225	veliparib +TMZ					2 m	17.00%	10.3 m	~38%

^1^When exact percentages and survival times were not provided, these were estimated from the time to progression and survival curve.

## nRTK and Intracellular Signal Transduction Pathways

Recently, nRTKs have gradually come into the field of view of researchers. Approximately 32 nRTKs have been identified in human cells, such as the SRC family, FAK family, and ABL family. Most of them are located in the cytoplasm, with part of them anchored on the cellular membrane by amino-terminal modification (73, 74). At first, the main function of nRTKs is associated with the immune system, such as activation of T and B cells (75). Despite lacking an RTK-like extracellular ligand-binding domain, nRTKs possess an ATP-binding site and a tyrosine kinase catalytic domain. So nRTKs can function partly as the same as transmembrane tyrosine kinases to regulate cell growth, proliferation, differentiation, adhesion, migration, and apoptosis. Beyond that, as nRTKs are located in the cytoplasm, they can bind proteins, lipids, and DNA through different domains to act broadly. Small-molecule inhibitors play a dominant role in targeted therapy for nRTKs instead of monoclonal antibodies due to their intracellular location and structure. In addition, some proteins functioning as part of the intracellular signal transduction pathway, such as Akt, MEK, and Erk, are also targets of therapy and discussed in this part.

Until June 2021, there are 48 clinical studies (20 for newly diagnosed GBM and 28 for progressive GBM) involving 9 small molecules published in PubMed. The radiological response rates are optimistic but not mTOR inhibitors compared to baseline. Thus far, the PFS and OS have not shown obvious benefits (**Additional File 1**).

### Src

Since Francis et al. found the first proto-oncogene v-Src in the Rous sarcoma virus, 11 members of the human Src Kinase family (SFK) have gradually been discovered (76). Among these, c-Src, Yes, Fyn, Lyn, and Lck have functional roles in glioma involved in survival, proliferation, migration, angiogenesis, irradiation therapy resistance, and even stemness maintenance (77–81). Src kinase is the first characterized proto-oncogene (79, 82). The Src Kinase family thus likely functions as a traffic node of a complicated gene regulatory network initiated by membranes such as growth factor receptors, G protein-coupled receptors, and cytokine receptors (83, 84). Then, SRC-family kinases cross-talk with multiple pathways and are involved in the regulation of FAK/STAT3, Wnt/beta-catenin, caspase, cyclin/CDK, and integrin/FAK intracellularly (85–87). In GBM, the activity of Src kinase was significantly increased even though there was little missense mutation or amplification (88). Whereas mutation or loss of PTEN and EGFRvIII mutation would activate Fyn and c-Src, respectively, SRC-family kinase activity increasing is due to upstream deregulation instead of SRC *per se* (23, 78, 89). CD90 high-expression cells, which are identified as glioma stem cells, are sensitive to Src inhibitors (77). Moreover, Src activation and downstream multiple RTKs partially account for radiotherapy resistance. Si306, an inhibitor of c-Src, can increase the sensitivity to radiotherapy (79). To date, dasatinib and ponatinib have entered clinical trials in the area of glioma. These drugs also belong to pan-TKI.

Dasatinib is a second-generation TKI that can inhibit many nRTKs, including all types of kinases of the SRC family, such as c-Src, Lyn, Fyn, and Yes, which play important roles in the brain. Simultaneously, in a high concentration, it can impair other kinase activities, including BCR-ABL, Eph A2, c-kit, and VEGFR2, particularly PDGFR, which is altered in most diffuse intrinsic pontine gliomas (90, 91). To date, dasatinib has been evaluated along with conventional treatments or other TKIs in phase I and phase II clinical trials for GBM and diffuse pontine glioma in children and adults. The safety and tolerability of dasatinib demonstrated in the studies is disappointing, particularly when combined with lomustine in recurrent GBM and in combination with crizotinib in children with glioma (92, 93). Although theoretically anti-Src therapy can suppress radiation resistance, in a phase II trial involving 217 patients with newly diagnosed GBM, and the combination of dasatinib with concomitant radiation and TMZ did not improve PFS and OS compared with placebo (NCT00869401). Bev. and dasatinib in combination can numerically increase the PFS but not OS compared with Bev. alone (NCT00892177) (94). The above results suggest that it is effective to combine dasatinib with TKIs or various targeted drugs at the level of cellular mechanisms, such as c-MET, PDGFR, and VEGFR inhibitors (95, 96), but useless in clinical trials.

Ponatinib, the third-generation TKI for CML, targets Src and VEGFR, PDGFR, and FGFR, which are three crucial receptors associated with angiogenesis. Therefore, ponatinib is a potent anti-angiogenesis drug. It did not show a promising effect in a phase II study including 15 patients with Bev.-refractory GBM (97).

Bosutinib is a small molecule inhibitor targeting BCR-ABL and Src and is used for treating CML. In 2014, a phase II study involving only 11 patients with recurrent GBM showed a better mOS of 11.7 m compared with baseline. More clinical trials are warranted to evaluate the efficacy of bosutinib (98).

### PKC

Protein kinase C is a serine/threonine kinase that includes three subgroups: the classical isoforms, novel isoforms, and atypical isoforms. Among the subgroups, there are tens of isoforms functioning differently in the survival, proliferation, adhesion, migration, and therapy resistance of glioma cells because of the diversity of phosphorylation sites, types of stimuli, and cell environment (99). The traditional signal pathway is the PLC/PIP2/IP3 pathway. But studies in various cancers, including GBM, showed that overexpression of PKC contributes to tumor pathogenesis and seems to be involved in EGFR, PI3K/Akt, Ras/MEK/MAPK, hedgehog, and TNFα signaling, which are deregulated in GBM and are deemed to be potent targets. In addition, PRKD2, a member of the PKC-activated protein kinase family, was identified as a mediator of GBM growth involving decreasing p53 and regulating the phosphorylation of retinoblastoma protein (100). RTKs, p53, and Rb, the three pathways that are commonly deregulated in GBM, crosstalk with PKC (101). Therefore, PKC inhibition is an important approach for the treatment of GBM and many trials test its efficacy. Targeting atypical PKC decreases tumor growth in EGFR inhibitor-resistant mouse models of GBM (102). PKCδ, an isoform of novel PKC, acts as a critical mediator of the maintenance of tumor stem cells through an autocrine loop with positive feedback that is driven by the PKCδ/STAT3/IL-23/JAK signaling axis (103).

Tamoxifen is a selective estrogen receptor modulator used to treat and prevent breast cancer in women (104). Strictly speaking, tamoxifen is not a classical small-molecule inhibitor. In GBM, it functions as a targeted inhibitor of PKC. Because overexpression of PKC in GBM is associated with TMZ and irradiation resistance and tamoxifen has no significant overlapping toxicities with most other drugs, clinical trials combine tamoxifen with traditional therapies for newly diagnosed GBM. Up until now, there have been no definite results for the regimen in phase II trials. A phase II trial of RTOG protocol BR-0021 combining high-dose tamoxifen with radiation did not exhibit improved effects (105). The later trials combining tamoxifen with TMZ and RT also did not have a different outcome.

Enzastaurin is a synthetic bisindolylmaleimide with potential antineoplastic activity. Enzastaurin binds to the ATP-binding site and selectively inhibits PKC β, a classical isoform involved in the induction of VEGF-stimulated neo-angiogenesis. In glioma, enzastaurin functions as an antiangiogenesis drug, entering clinical trials (106). In 2010, enzastaurin entered a phase III study as monotherapy but showed modest efficacy compared with the control arm of lomustine or baseline (107).

### PI3K/Akt/mTOR

PI3K/Akt/mTOR, including the endogenous inhibitors PTEN, TSC1/2, and PHLPP, is one of the most common and most characterized malfunction pathways in glioma (108). High activity in the pathway, in general, portends a poor outcome (109). Since Powis et al. proposed that inhibition of PI3K by wortmannin could repress growth of human tumor xenografts in mice (110), there have been more than 20 types of small-molecule inhibitors entering clinical trials and more than 50 types tested in laboratories. In addition, many inhibitors can regulate the activity of PI3K/Akt to inhibit tumors, though they do not immediately target the pathway, such as oxymatrine, baicalin, and gartanin (111–113). Primarily, targeted therapy for PI3K/Akt/mTOR is mainly proposed to solve the resistance of chemoradiotherapy (114). On the one hand, the pathway is the busiest road of signal transduction that is initiated by growth factors or known as various RTKs and G protein-coupled receptors (GPCRs) on the cytoplasm membrane. On the other hand, it can crosstalk with diverse pathways intracellularly, such as Ras/Raf, Notch, STAT3, and Wnt/catenin. All of the proteins in the pathway, and upstream RTKs, and the endogenous inhibitors of the pathway are usually detected malfunctioning in glioma (114). Whereas in this pathway, every protein is an independent target, there is a combination regimen targeting more than one protein to increase efficacy. Simultaneously, PI3K has four classes and various isoforms with different catalytic-subs which play different roles. Among them, class I is dominant in cancers (114, 115). Therefore, there are pan-PI3K, isoform-selective, and dual PI3K/mTOR inhibitors. The common mutation of PIK3CA (encodes p110α, a 110 kDa catalytic subunit of Class IA) in cancer causes constitutive activation of PI3K and downstream Akt, and it is perhaps a promising target for therapy, but the mutation is not common in glioma despite 90% of GBMs harboring deregulated PI3K (116, 117). Other isoforms also impact the pathogenesis of solid tumors and hematologic malignancies. For instance, PTEN-deficient GBM largely depended on p110α for proliferation and p110β for migration (118). Recently researchers found many inhibitors can increase reactive oxygen species (ROS) to regulate the ROS-JUN-p53 loop and then decrease the level of phosphorylation and activity of PI3K/Akt/mTOR (119, 120). Inhibition of PI3K would promote the expression of the genes associated with glioma stem cells, such as SOX2, OCT4, and MSI1 (121). This is probably one of the mechanisms of resistance.

Akt, also known as PKB, includes three isoforms, and among these, Akt1 plays a major role in the PI3K/Akt pathway (122). It is not only an indispensable part of the PI3K/Akt pathway but also a serine/threonine-specific protein kinase that activates NF-κB, Bcl-2 family protein, and MDM2 to regulate multiple cellular processes (123). In addition, Akt also accepts the immediate regulation driven by other kinases (124). The drugs, which target Akt, combined with TMZ and fractional radiation, gradually enter the field of vision because chemoradiotherapy would increase the level of phosphorous Akt (125, 126).

Lastly, targeting mTOR is the most studied approach for the treatment of glioma. There are two different complexes, regulatory-associated protein mTORC1 and rapamycin insensitive mTORC2, the latter functioning in resistance to rapamycin by activating Akt in a positive feedback manner (127). 2-hydroxyglutarate (2HG) produced by IDH1/2 mutation promotes mTOR activity by depleting KDM4A and decreasing DEPTOR protein stability (128), presenting another mechanism of IDH1/2 mutation promoting the genesis of glioma and the possibility of a combination IDH inhibitor and mTOR inhibitor. Simultaneously, the pathway is a hotspot of studies on microRNA, lncRNA, and exosomes because of the extensive functions of noncoding RNA and PI3K/Akt (129–132). In addition, the downstream targets of the pathway, such as p70S6K, 4EBP1, and eIF-4E, are also druggable targets (133). However, in summary, the approach of targeting PI3K/Akt is failing in glioma clinical trials (**Additional File 1**). Single-target drugs are usually fed back to activate upstream and other pathways such as Erk through complex networks (124, 133). The content of phosphorous Akt, the status of PTEN, and other proteins may be prognostic factors that indicate the sensitivity to PI3K/Akt inhibitors (134, 135), but to date, the tissue analysis usually does not show that the molecular markers correlate with survival. The occurrence of heterogeneity and stem cells within the tumor adds to these difficulties (121).

### PI3K

As mentioned above, there are isoform-selecting and pan-PI3K inhibitors. The former includes eganelisib, idelalisib, and alpelisib, but does not enter clinical trials in glioma. Eganelisib is a highly selective inhibitor of the enzyme PIK3C gamma (p110gamma) (136). But p110 gamma is predominantly expressed in the pancreas, skeletal muscle, liver, and heart instead of the brain (137). Idelalisib, approved by the FDA in 2014, blocks selectively P110δ, which is expressed in normal and malignant B-cells. Alpelisib is a PI3K alpha specific inhibitor for the treatment of breast cancer with a PIK3CA mutation after disease progression (138).

The drugs targeting PI3K that have entered clinical trials are mainly Pan-PI3K inhibitors, namely, taselisib, pilaralisib, buparlisib, and copanlisib. Thus far, the trials are mainly MATCH (Molecular Analysis for Therapy Choice) models because PI3K has diverse effects on the regulation of biological processes in a wide variety of human cancers (139). Taselisib is a pan-PI3K inhibitor but has the strongest activity to repress p110α (140, 141). Therefore, it is used in cancers with PIK3CA mutations, in particular uterine serous carcinomas and breast cancers, which are accompanied by hormone receptor changes because of the cross-talk between estrogen receptor (ER) and PI3K (141). The relationship between the expression of estrogen receptors and glioma expression has gained attention recently because of sex-specific differences in GBM (142). Copanlisib is predominantly against PI3K-α and PI3K-δ isoforms (143). A phase II MATCH trial involving the above two pan-PI3K inhibitors is recruiting (NCT02465060).

Pilaralisib, another pan-PI3K inhibitor, has been tested in early clinical trials. A phase I trial indicated that pilaralisib had a favorable safety profile (144), although some researchers thought that the toxicity of pan-PI3K was high (141).

Buparlisib is the most studied pan-PI3K inhibitor in glioma. It presented a promising prospect in preclinical studies. At low concentrations, buparlisib can inhibit the migration and invasion of GBM cells, and at higher concentrations and with a longer duration of drug exposure, it can promote apoptosis of cells (145, 146). It is controversial how the status of PTEN influences the efficacy of buparlisib. Koul showed that PTEN and EGFR status did not correlate with sensitivity, but mutation or wild type of p53 could change the method of death of tumor cells after treatment with buparlisib (147). But Xie et al. found that the buparlisib activity of anti-p110beta was poor and that may limit the efficacy of treating PTEN-deficient GBM (118). Buparlisib was also combined with other targeted therapies such as HSP990 (an Hsp90 inhibitor) and ABT-737 (a Bcl-2 inhibitor) because inhibiting the proteins in other pathways would cause compensatory overactivation of PI3K/Akt (148, 149). Yet, buparlisib is combined with other therapies is in phase I or phase II clinical trials (150–152). To date, the safety profile of buparlisib is deemed poor, and the regimen did not show enough efficacy to improve the outcome.

### Akt

Akt inhibitors, including ipatasertib, capivasertib, MK2206, afuresertib, and perifosine, thus far are less studied in glioma. Ipatasertib is currently in phase II trials for the treatment of solid tumors. Capivasertib and afuresertib have mainly been tested in preclinical studies. MK2206 acts as an allosteric Akt inhibitor and is a highly selective inhibitor of pan-Akt, namely, of all three Akt isoforms Akt1, Akt2, and Akt3 (153). MK2206 can induce apoptosis and autophagy, increasing the efficacy of gefitinib (154). Narayan et al. showed that MK2206 at a low concentration (1 μM) reduced the phosphorylation of Thr308 and Ser473 residues of AKT in both adherent GBM cells and spheroids to sensitize them to irradiation and TMZ. At a high concentration (>5 μM), it inhibited invasion and migration of cells (155). But in PTEN-deficient cells, MK2206 could not decrease the level of phosphorylation of mTOR and S6K, the effectors of the PI3K/Akt pathway, nor induce apoptosis or autophagy.

### mTOR

mTOR inhibitors are the most studied small molecules in the pathway. Now there are three generations of inhibitors and more than 70 clinical trials. Everolimus, a generation I mTOR inhibitor, is a medication used as an immunosuppressant to prevent rejection of organ transplants and in the treatment of renal cell cancer and other tumors, approved by the FDA for various conditions. Everolimus is more selective for the mTORC1 protein complex (156). It binds to its protein receptor FKBP12, which directly interacts with mTORC1 with little impact on the mTORC2 complex (156). Besides, everolimus could inhibit the production of glutathione from glutamine and glutamate and then inhibit the repair of DNA damage caused by carboplatin (157). But everolimus can lead to a hyper-activation of the kinase Akt *via* inhibiting the mTORC1 negative feedback loop while not inhibiting the mTORC2 positive feedback to Akt. And it has been evaluated in GBM for more than a decade with 26 trials registered on ClinicalTrials.gov (158). But a randomized phase II clinical trial RTOG 0913 (NCT01062399) presented a disappointing result: that adding everolimus into standard therapy reduced mOS by 4.7 months relative to the control arm (159). This leads us to consider that the penetration through the BBB, the toxicity of everolimus, and the activity of mTORC2 are influential but uncontrollable (158). But the next two early phase studies in pediatric patients demonstrated the manageable toxicity profile of everolimus (160, 161). On this basis, we think the prospect of mTOR inhibitors is promising. The PI3K/Akt/mTOR is a busy traffic node in cells, hence it is an important target just like RTKs and Ras/Raf/MAPK. The combination of mTOR inhibitors and targeted inhibition of Ras and PDGFR α is still a possible way to treat glioma (160, 162). The dual inhibitor of mTORC1 and mTORC2 may be a vulnerability in cancer (163). In addition, everolimus plays an irreplaceable role in the treatment of tuberous sclerosis complex and is mainly used for neurofibromatosis type 1-associated pediatric low-grade glioma and subependymal giant cell astrocytoma.

Sirolimus, also known as rapamycin and initially developed as an antifungal agent, functions as an immunosuppressive small-molecule inhibitor by inhibiting activation and sensitivity to interleukin-2 (IL-2) of T and B cells through inhibition of mTOR (164). Although it is a generation I inhibitor of mTOR like everolimus, it can also inhibit mTORC2. However, this has the adverse effect of increasing the risk of type 2 diabetes (165). The effect of sirolimus is complex, even though many studies have indicated that sirolimus can enhance mouse lifespan in a sex-specific manner (166). Temsirolimus, a derivative and prodrug of sirolimus, is converted to sirolimus (rapamycin) *in vivo*. But temsirolimus also shows exclusive activity on its own, and the trials in glioma mainly use temsirolimus instead of sirolimus. Outcomes of temsirolimus in clinical trials are frequently worse than those in the control arm. The combination of temsirolimus and TKIs, such as erlotinib and sorafenib, limits the dose of temsirolimus to one-tenth of MTD so as to achieve acceptable safety because of the side effects of temsirolimus. Thus, the regimen should be tested in more studies combining mTOR inhibitors and TKIs (37, 167, 168). Combining temsirolimus and perifosine, an Akt inhibitor, theoretically inhibits mTORC1 and suppresses the activity of Akt, which is activated by mTORC2 in a back-feed manner. The regimen was tolerable in a phase I study (169).

### Ras/Raf/MEK/Erk

Ras/Raf/MEK/Erk is another busy traffic road in cells in addition to PI3K/Akt. Firstly, Ras is a protein superfamily of small GTPases and, in general, is responsible for cell proliferation (170). Ras is one of the most frequently altered proteins in cancer, but rarely in glioma (171). However, it is not a common target of therapy for cancer despite the many efforts contributed to the relative studies (172). Raf kinases are a family of three serine/threonine-specific protein kinases, including A-Raf, B-Raf, and C-Raf. BRAF became the focus of research recently since a large portion of human tumors carry oncogenic ‘driver’ mutations in the BRAF gene, with 11% of glioma cell lines harboring BRAF mutations (171, 173). The activating mutation in BRAF is a common change in pediatric low-grade gliomas (174). The BRAF V600E mutation inducing the constitutive activity of Raf is frequently detected in low-grade pleomorphic xanthoastrocytoma, ganglioglioma, extra-cerebellar pilocytic astrocytoma, and epithelioid GBMs instead of other types of high-grade glioma (175). But the overactivation of Ras/Raf is highly frequent in GBM. The BRAF V600E mutation is a successful target in melanoma, non-small cell lung cancer, and thyroid cancer. In high-grade gliomas, it may become a biomarker of benefit from dabrafenib (BRAF inhibitor) and trametinib (MEK inhibitor) dual-targeted therapy (176). MEK is also known as MAPKK, and Erk is a classical MAPK, a classical cascade amplification pathway. The pathway connects extracellularly initiated signals from receptors on the surface of cells to the DNA in nuclei to regulate multiple biological processes. In addition, MEK and MAPK can function as kinases to immediately phosphorylate other proteins. Deregulation of the MAPK/Erk pathway is a necessary step in the malignant transformation of many cancers.

In summary, the intracellular signal transduction pathway is a significant embodiment of the complexity of biological regulation. On the one hand, every kinase can accept regulation from upstream kinase and activate downstream kinase to form a complicated network. This network is critical for maintaining the rule cell behavior. However, the overactivation of the pathway is usually due to upstream RTKs and GPCRs but not the abnormality of proteins comprising the pathway. Therefore, we suppose that it is not an ideal target for tumor treatment. Perhaps, targeting the intracellular signal transduction pathway would play an assistant role if the tumor showed significant overactivation or TKIs caused the second pathway overactivation by tissue analysis. The utilization of these inhibitors still requires biomarkers for precision medicine.

## Proteins Regulating Cellular Biological Processes

In addition to the kinome, there are several proteins regulating cellular biological processes. The small molecules inhibiting the proteins are usually highly selective but difficult to target. There were 11 small molecules that were involved in 20 clinical trials that were published in PubMed (**Additional File 1**).

### HDAC

Epigenetic changes refer to alternations affecting the expression of genes and the phenotypes of cells but not changing the sequence of DNA. It plays an important role in multiple aspects of pathogenesis and therapy resistance (177). In general, post-translational acetylation of histones modulates the structure of chromatin by relaxing the latter to promote the binding of transcription factors with motifs in genes and then increase the expression level of tumor suppressor genes (178). In addition, GSCs have a distinct pattern of epigenetic alterations, including DNA methylation and histone modifications (13). The process is controlled by the balance between the histone acetyltransferases (HATs) and histone deacetylases (HDACs). There are at least four classes of HDACs, including various isoforms (179). Different isoforms function in various ways. This indicates that HDAC inhibitors targeting multiple isoforms may be more effective (180–184). Class I and class II were studied more thoroughly, and the disruption of HDACs was observed in multiple cancers, particularly diffuse intrinsic pontine gliomas with H3K27M mutations (182, 185–187). HDAC inhibitors decondensing chromatin can prevent DNA double-strand break repair and regulate the stemness of GBM cells to induce radio sensitization (180, 188, 189). HDAC inhibitors also regulate the post-translational acetylation of proteins in the cytoplasm to influence the function of the latter, which is involved in angiogenesis, stemness, immune regulation, and ultimately inducing apoptosis. For example, many studies suggested that HDACs could downregulate the activity of p53, a classical tumor suppressor protein (185, 190, 191). HDACs block the NF-κB pathway by acetylation to inhibit resistance to TMZ and radiation (192, 193). But there was a study that indicated HDAC inhibitor suberoylanilide hydroxamic acid (SAHA) favored the acquisition of TMZ resistance by increasing recruitment of SP1, C-JUN, NF-κB, and p300 within the relaxing MGMT promoter region (194). HDAC inhibitors repress EGFR/EGFRvIII expression inducing the expression of FOXO1—a tumor suppressor—in MYC-driven medulloblastoma cells (195, 196). HDACs also influence the polarization of microglia mediated by glioma cells (197). In addition, Nguyen et al. found that HDAC inhibitors blunted glycolysis in a c-Myc dependent manner and lowered ATP levels to elicit metabolic reprogramming to dependence on fatty acid oxidation (198). In summary, HDACs impact multiple processes through epigenetic changes. In the past few years, there have been a number of studies in which HDAC inhibitors were combined with radiation, chemotherapy, and other targeted drugs, presenting exciting efficacy *in vitro* and *in vivo* (192, 199–201). Because it is pharmacologically much simpler to inhibit an enzyme than to induce one, HDAC inhibition has gained enormous clinical interest as an anticancer strategy (177). At present, HDAC inhibitors as anti-tumor drugs are mainly used for the treatment of hematological neoplasms. Some HDAC inhibitors, such as valproic acid, have been used as anti-seizure drugs in GBM (202). To date, besides vorinostat, entinostat, Panobinostat, and VPA, many new HDAC inhibitors are still found and being studied preclinically. Interestingly, HDAC inhibitors are also being studied for their potential to induce viral HIV-1 expression in latently infected cells and disrupt latency (203).

Vorinostat, also known as SAHA, binds to the active sites of HDACs and acts as a chelator for zinc ions, which are also found in the active sites of HDACs. It acts on classes I, II, and IV of HDAC (204). In addition, SAHA could trigger autophagy in GBM stem cells through the Akt/mTOR pathway. Many clinical trials are ongoing or completed, but most are phase II or earlier. Based on preclinical results, vorinistat-combined drug with chemoradiotherapy, Bev., or bortezomib (a proteasome inhibitor) (205, 206). In a phase II north central cancer treatment group study involving 66 patients with recurrent GBM, vorinostat monotherapy showed nice tolerability but modest drug activity (207), and a phase I/II trial of vorinostat combined with traditional therapy for newly diagnosed GBM presented the same result. Because of the compromised efficacy presented in recent trials, there are additional trials registered in NIH except for a phase I trial of pembrolizumab and vorinostat combined with temozolomide for newly diagnosed GBM. Now preclinical studies focus on molecule markers such as IDH1 mutation, pChek2, and Bcl-XL, imaging parameters, which indicate sensitivity to vorinostat and other HDAC inhibitors (208–210), or to identifying some targeted inhibitors, such as melatonin, chloroquine, and gamitrinib, which can function as synergic effects with HDAC inhibitors (201, 211).

Romidepsin, also known as FK228 or Istodax, is a natural product obtained from bacteria. It acts as a prodrug, with the disulfide bond undergoing reduction within the cell to release a zinc-binding thiol. Thiol binds to a zinc atom in the binding pocket of Zn-dependent HDAC to block its activity. So, in theory, it acts on HDACs of the zinc-dependent classes I, IIa, IIb, and IV (179). Romidepsin decreases the expression of p21 to induce apoptosis (212). Wu et al. found that romidepsin increased the sensitivity to TMZ by blocking the PI3K/Akt/mTOR pathway (213). A phase I/II trial of romidepsin for adults with recurrent malignant glioma failed (214).

Panobinostat, trade name Farydak, is a hydroxamic acid and acts as a non-selective HDAC inhibitor. There are plenty of studies to research the activity in the treatment of diffuse intrinsic pontine glioma, a lethal pediatric brain cancer usually harboring mutations altering the epigenetic regulatory histone tail (H3 K27M) (215). Panobinostat can increase H3 acetylation and H3K27 trimethylation to partial rescue of the H3K27M-induced global hypotrimethylation phenotype (216). At present, Panobinostat is being tested in phase I clinical trials for diffuse intrinsic pontine glioma and GBM. Its potential of anti-angiogenesis led to combination with Bev. but failed in phase II (217).

Valproic acid is presently extensively used for epilepsy, bipolar disorder, and migraine headaches. In addition to inhibiting HADCs, it can affect GABA levels and voltage-gated sodium channels (218). Retrospective trials indicated that patients with GBM receiving valproic acid during the traditional regimen of TMZ and radiation had an improved prognosis by sensitizing to chemoradiotherapy (219–222). But Berendsen et al. proposed that epileptogenic GBM is a favorable prognostic factor regardless of using antiepileptic drugs or not (223). In 2016, a retrospective trial including 1,869 patients showed that valproic acid or levetiracetam, another anti-epileptic drug, did not improve the outcome of newly diagnosed GBM (224). To date, the activity of valproic acid in GBM is controversial. A phase II trial involving 43 patients with high-grade gliomas who received valproic acid and TMZ plus radiation indicated no increase in mOS (NCT00302159).

Belinostat, trade name Beleodaq, previously known as PXD101, is a hydroxamate-based pan-HDAC inhibitor that induces apoptosis by upregulation of p21 and through multiple pathways just like other HDAC inhibitors (225). There is currently no glioma clinical trial registered with the NIH.

### CDK4/6

Deregulation of cyclin‐dependent kinase 4/6 (CDK4/6) is detected in various types of cancers, including glioma (226). Targeted inhibition of CDK4/6 has emerged as an efficient approach for treating breast cancer, with three small-molecule inhibitors approved by the FDA: ribociclib, palbociclib, and abemaciclib (227). CDK4/6 inhibitors primarily arrest the cell cycle in the G1 phase by regulating the CDK4/6–retinoblastoma (Rb)–E2F pathway to inhibit proliferation. However, as more and more studies are conducted *in vitro* and *in vivo*, the new targets of CDK4/6 inhibitors and other pathways involved in CDK4/6 have been proposed, involving cellular metabolism, autophagy, and immune evasion, despite the high selectivity of third-generation CDK4/6 inhibitors (228). The on-target and off-target mechanisms of CDK4/6 inhibitors have been reviewed by Denisa et al. (227). According to the TCGA database, the CDK4/6-Rb-E2F axis is deregulated in about 80% of GBMs, including endogenous inhibitors of CDKs, such as p16, an INK4 family protein (229). A large-scale data analysis on comprehensive genomic profiling indicated that 47.1% of brain gliomas harbored at least one cyclin alteration (230). In glioma, the activity of CDK4/6 inhibitors depends on the wild type of Rb. Mutation or deletion of Rb causes resistance to the drug. The deficiency of endogenous inhibitors, such as p16 and p18, or known as CDKN2A and CDKN2C, is a strong predictor of sensitivity to CDK4/6 inhibitors (231, 232). Besides, SHH and MYC amplification of group 3 medulloblastoma is sensitive to inhibition of CDK4/6 (233). The RTKs or growth factor pathway, p53, and CDKs/Rb/E2F pathways are the three most characterized pathways involved in the pathogenesis of glioma (234). Previous studies found that the combination of CDK4/6 and RTK inhibitors, such as mTOR inhibitors or c-Met/Trk inhibitors, could play a synergy (229, 235), possibly because the monotherapy of TKIs or CDK inhibitors would activate each other. Simultaneously, inhibition of CDK4/6 can delay radiation resistance and TMZ resistance by repressing double-strand break repair (236, 237). In general, the trials of CDK4/6 inhibitors are mainly in the early phase.

Palbociclib is the first and most studied CDK4/6 inhibitor in glioma therapy. But acquired resistance is still a problem that urgently needs to be addressed (238). On the one hand, the deregulation of the CDKs/Rb/E2F axis is a source of resistance to palbociclib. For example, gliomas with deletion or mutation of Rb are naturally insensitive to CDK4/6 inhibitors (232), but the wild-type Rb cancers would overexpress the Rb protein to gradually promote resistance to palbociclib (239). In addition, the isoforms of cyclins and CDKs, such as CDK2 and cyclin E, can play compensatory roles (227). However, a study found that palbociclib only induced reversible quiescence but not irreversible senescence in glioma stem cells (240). Thus far, palbociclib entered phase II trials for refractory or recurrent GBM, but for the moment it did not show enough efficacy to improve the outcome.

Ribociclib is used along with an aromatase inhibitor (such as letrozole). Some studies indicated the good CNS penetration of ribociclib (241). Ribociclib demonstrates a synergistic effect when combined with ALK or MEK inhibitors in the treatment of neuroblastoma (242). A phase I/II study of ribociclib following radiation therapy in children with newly diagnosed diffuse intrinsic pontine glioma indicated that the regimen was feasible (243).

Based on CDK4/6 inhibitor suppressing immune evading, the regimen of abemaciclib plus cancer immunotherapy, such as humanized antibody targeting the programmed cell death protein 1 (PD-1) receptor of lymphocytes pembrolizumab, has entered clinical trials. Thus far, there has been no result.

### PARP

Poly (ADP-Ribose) polymerase (PARP) is a family of enzymes that catalyze the synthesis of linear or branched polymers of ADP-ribose (PAR) using NAD+ as substrate, and functions as radiosensitizer (244). Once PARP detects single-strand DNA breaks, it binds to DNA through the DNA-binding domain to induce structural change. Then it initiates the synthesis of a polymeric adenosine diphosphate ribose chain. The auto-PARylation of PARP on the auto-modification domain is a signal that recruits DNA ligase III (LigIII), DNA polymerase beta (polβ), and scaffolding proteins such as X-ray cross-complementing gene 1 (XRCC1) to repair the damaged DNA, a process known as base excision repair (BER) (245). Poly (ADP-ribose) glycohydrolase (PARG) degrades the PAR chain to recycle the PARP. Thus far, there are 18 members in the family, but PARP1 and PARP2 play major roles in repairing DNA, especially the first one. Beyond that, the other members perform other functions, and the PARylation of proteins as an important and ubiquitous post-translational modification involving histones and various transcription factors can also regulate multiple processes of cells, such as caspase-independent apoptosis by translating apoptosis-inducing factors into the nucleus (246–248). The high expression of PARP-1 was detected in multiple types of cancers (248). In the beginning, studies demonstrated that PARP inhibitors repressed the activity of PARP to hinder the BER system by competing with the substrate NAD+ (249). This would cause the accumulation of DNA damage of N-methylpurines (N7-methylguanine and N3-methyladenine) generated by TMZ and then eventually increase the sensitivity to TMZ and other alkylating agents. Notably, in the preclinical studies, the function of sensitization is still remarkable in MGMT-proficient and MMR-deficient glioma cells, which in general are regarded as drug-resistant cells (250–252). Higuchi et al. found that the TMZ sensitizer role of the PARP inhibitor was independent of base excision repair. There are perhaps other pathways by targeting which PARP inhibitors enhance the efficacy of chemoradiotherapy (253). For example, sustained inhibition of PARP-1 activity delays GBM recurrence by enhancing radiation-induced senescence (253). Similarly, PARP inhibitors can sensitize cells to ionizing radiation even in glioma stem cells, which can promote resistance to radiation by overexpressing PARP (254, 255), because the radiation generates single-strain DNA to exert clinical effect (256). Another important anti-tumor mechanism of PARP inhibitors is “synthetic lethality.” In some breast and ovarian cancers with BRCA mutations, meaning the deficiency of the homologous recombination repair system (HR), BER driven by PARP functions as compensation. So, the inhibition of PARP can maximize the effect of anti-tumor (257, 258). In glioma, deletion of PTEN, commonly occurring in primary GBM, can impact genomic stability by regulating the expression of RAD51, an important homologous recombination repair component (259). In *in vitro*, the cell lines with PTEN deficiency are more sensitive to PARP inhibitors than the wild-type PTEN cell lines (244). Inducing DNA damage is an important method to treat tumors. Except for chemotherapy and radiotherapy, topoisomerase inhibitors, topotecan and irinotecan, and inhibitors of DNA-dependent protein kinase (DNA-PK), a key enzyme involved in nonhomologous DNA end joining (NHEJ), can combine with PARP inhibitors to enhance the efficacy of antitumors (244). Recently, studies have demonstrated that IDH1/2 mutations could not only impair the HR system but also compromise the BER associated with PARP by decreasing NAD+ availability. This may be a reason for patients with IDH1/2 mutations have a better prognosis relative to IDH wild type when they receive chemoradiotherapy, and it also renders tumor cells sensitive to PARP inhibitors (260–262). Simultaneously, wild type p53, associated with cell cycle checkpoints and DNA repair, is supposed to indicate sensitivity to PARP inhibitors (263, 264). HDAC can repress DNA damage repair by epigenetic downregulation, which can play a synergistic role with PARP inhibitors (265). The inhibition of PARP can probably be used as an adjuvant therapy and there have been some clinical trials ongoing based on this theory. The loss of p53-binding protein 1 (53BP1), an antagonism factor of BRCA1, and the promoter of NHEJ can cause resistance to PARP inhibitors (266).

Olaparib, particularly in glioma harboring IDH mutation, the clinical trials were mainly carried out within recent 3 years, and there are no results thus far. OPARATIC trial, a phase I trial, demonstrated that olaparib could reliably penetrate recurrent GBM at radio sensitizing concentrations (267). Niraparib was tested in a phase II trial as a radiosensitizer (NCT04715620). Junko et al. showed that niraparib has the strongest potency in trapping PARP compared to olaparib and veliparib (268). In addition, pamiparib, talazoparib, and rucaparib which have been tested in other cancers.

In conclusion, the proteins regulating some specific cell processes, such as epigenetic changes, cell cycle, and DNA repair, may make a difference in glioma treatment. But in general, the relevant clinical trials are in their early phase. These inhibitors play functions by different mechanisms to have chance to combinate with TKIs in future clinical trials.

## Conclusion

Small-molecule inhibitors did not change the survival of GBM patients in clinical trials. But at present, it is not reasonable to define small-molecule inhibitors as a failure. On the one hand, most trials involving recurrent GBM would stop therapy of small molecules after the progression of the tumor. But the mPFS of recurrent GBM is only 2 months. It causes the duration of the small molecule therapies to be less than 2 months. The short exposure duration limits the efficacy of small molecules. In addition, the efficacy of small molecules includes relieving edema, improving of neurological function, and improvement of life quality. On the other hand, anatomical characteristics of the brain restrain the delivery and efficacy of drugs. Heterogeneity within the GBM mass, and specifically mosaicism of glioma stem cells, is a critical factor in maximizing efficacy. In clinical practice, the tailored therapy associated with molecular subtyping in different regions of a tumor mass and the change of recurrence is more useful than personal therapy. The time and space obstacles could be tackled by several methods, such as new approaches to drug delivery of drugs and the scheduling of drug administration. In the future, as the finding of new targets and new technologies for drug discovery replaces the “Me-too” model, small molecules will play a more functional role in the treatment of cancer and other diseases.

## Author Contributions

WH performed the selection of literature, drafted the manuscript, and prepared the figures and tables. DG designed this review, critically instructed the writing, and revised the manuscript. All authors listed have made a substantial, direct, and intellectual contribution to the work and approved it for publication.

## Funding

This project was supported by the National Natural Science Foundation of China (grant no. 81874086).

## Conflict of Interest

The authors declare that the research was conducted in the absence of any commercial or financial relationships that could be construed as a potential conflict of interest.

## Publisher’s Note

All claims expressed in this article are solely those of the authors and do not necessarily represent those of their affiliated organizations, or those of the publisher, the editors and the reviewers. Any product that may be evaluated in this article, or claim that may be made by its manufacturer, is not guaranteed or endorsed by the publisher.

## Supplementary Material

Additional File 1 Table S1.xlsx: A summary of outcomes in clinical studies in adult patients with glioblastoma.

Additional File 2 Table S2.xlsx: Some clinical trials being conducted.

## Abbreviations

GBM, glioblastoma multiforme; TCGA, The Cancer Genome Atlas; GSCs, glioma stem cells; NCCN, National Comprehensive Cancer Network; TMZ, Temozolomide; RT, Radiotherapy; Bev., Bevacizumab; EGFR, Epidermal growth factor receptor; OS, Overall survival; mOS, Median overall survival; PFS, Progressive free survival; mPFS, Median progressive free survival; OR, Response rate; PR, partial response; CR, complete response; SD, stable disease; RTK, Receptor tyrosine kinase; TKI, Tyrosine kinase inhibitor; nRTK, Nonreceptor tyrosine kinase; BBB, Blood–brain barrier; HDAC, Histone deacetylases; mTOR, mammalian target of rapamycin; PTEN, Phosphatase and tensin homolog; PARP, Poly (ADP-Ribose) polymerase; Grb,2Growth factor receptor-bound protein 2; GPCRs, G Protein-Coupled Receptors; NHEJ, Nonhomologous DNA end joining; HR, Homologous recombination repair system; BER, Base excision repair.
